# Supplementary figures and images for: An Introduced Crop Plant Is Driving Diversification of the Virulent Bacterial Pathogen Erwinia tracheiphila
Source: mBio. 2018 Oct 2;9(5):e01307-18. doi: 10.1128/mBio.01307-18 (PMC6168856; doi:10.1128/mBio.01307-18)

Supplemental Figure 1A

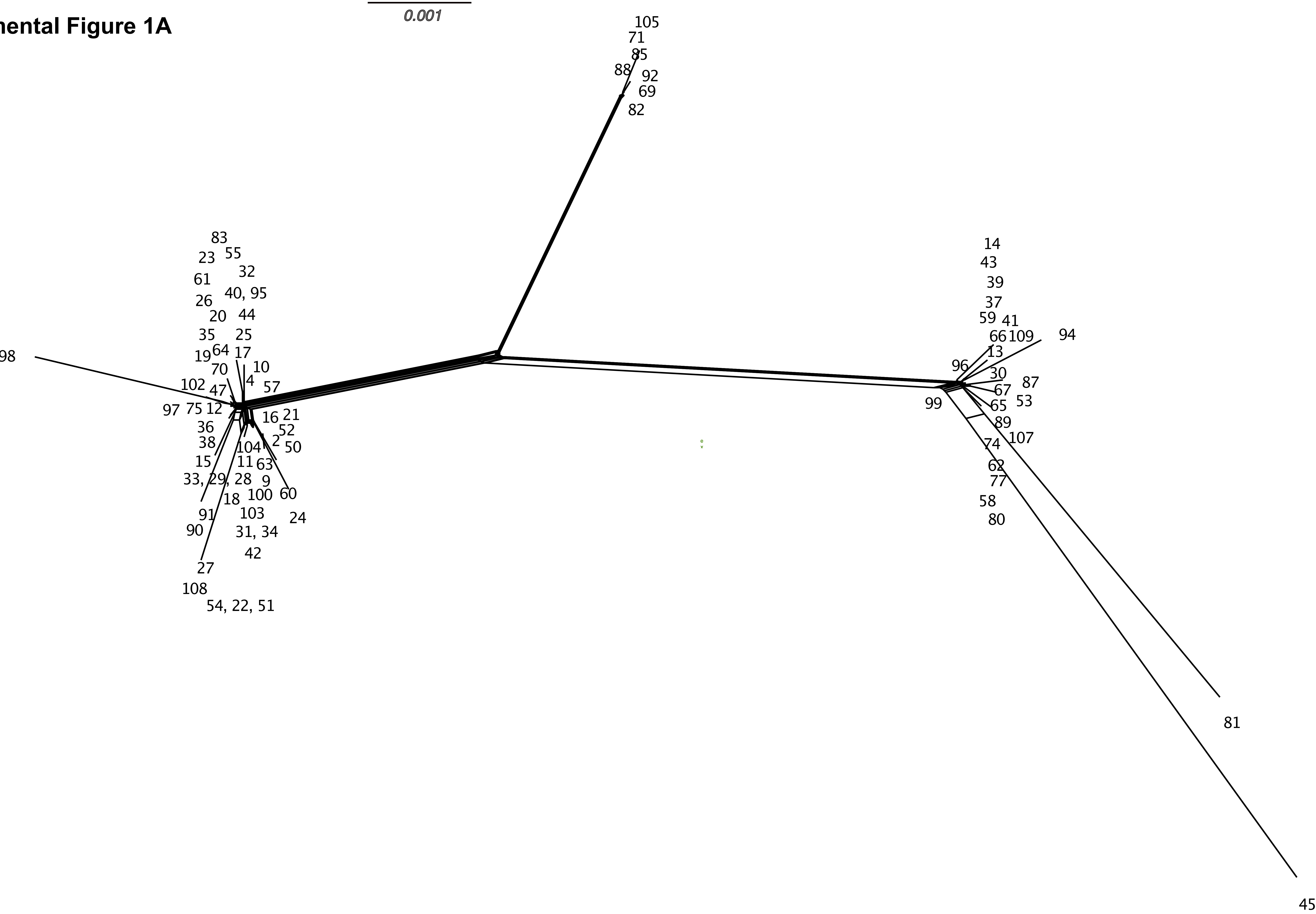

Supplemental Figure 1B

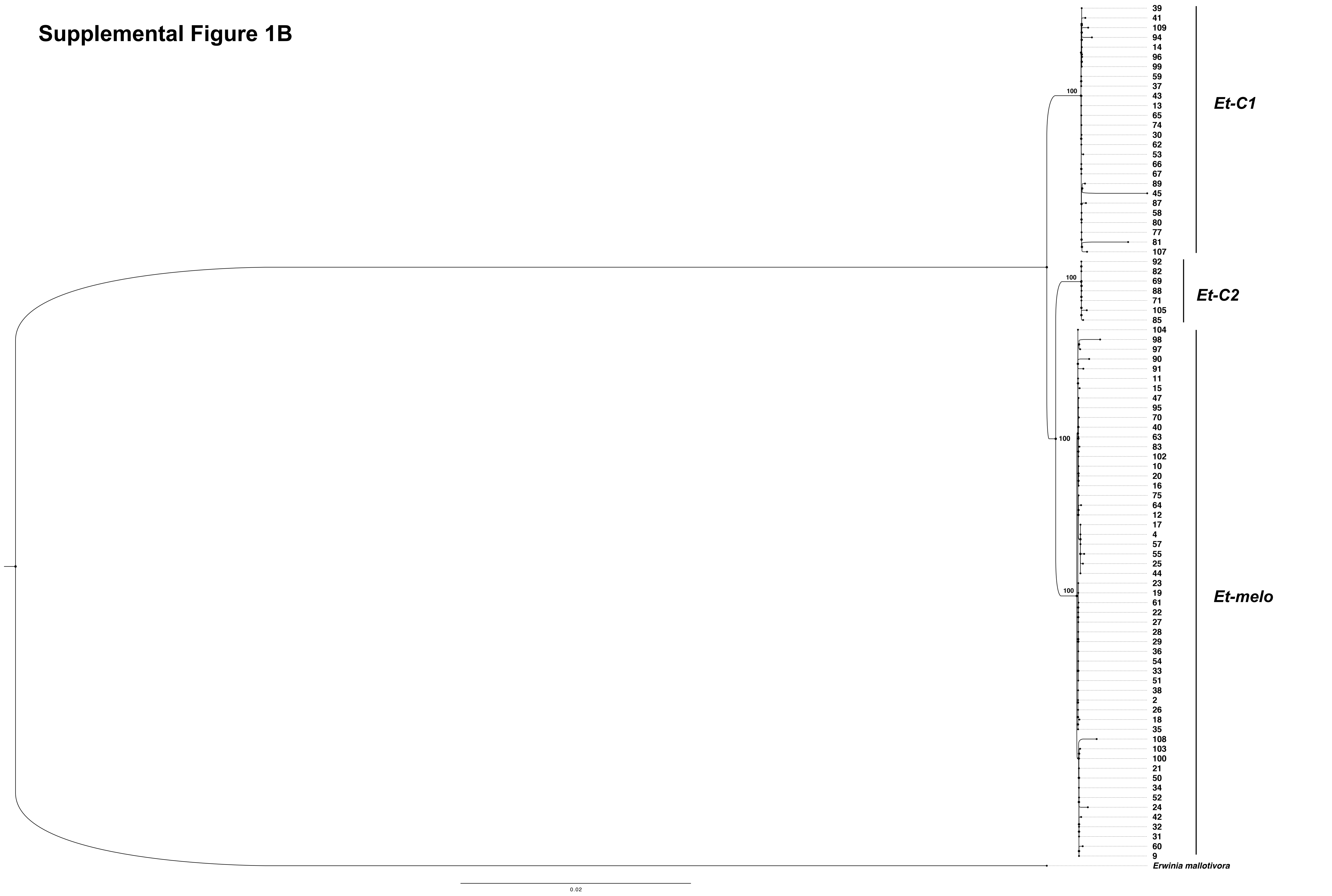

## Supplemental Figure 1C

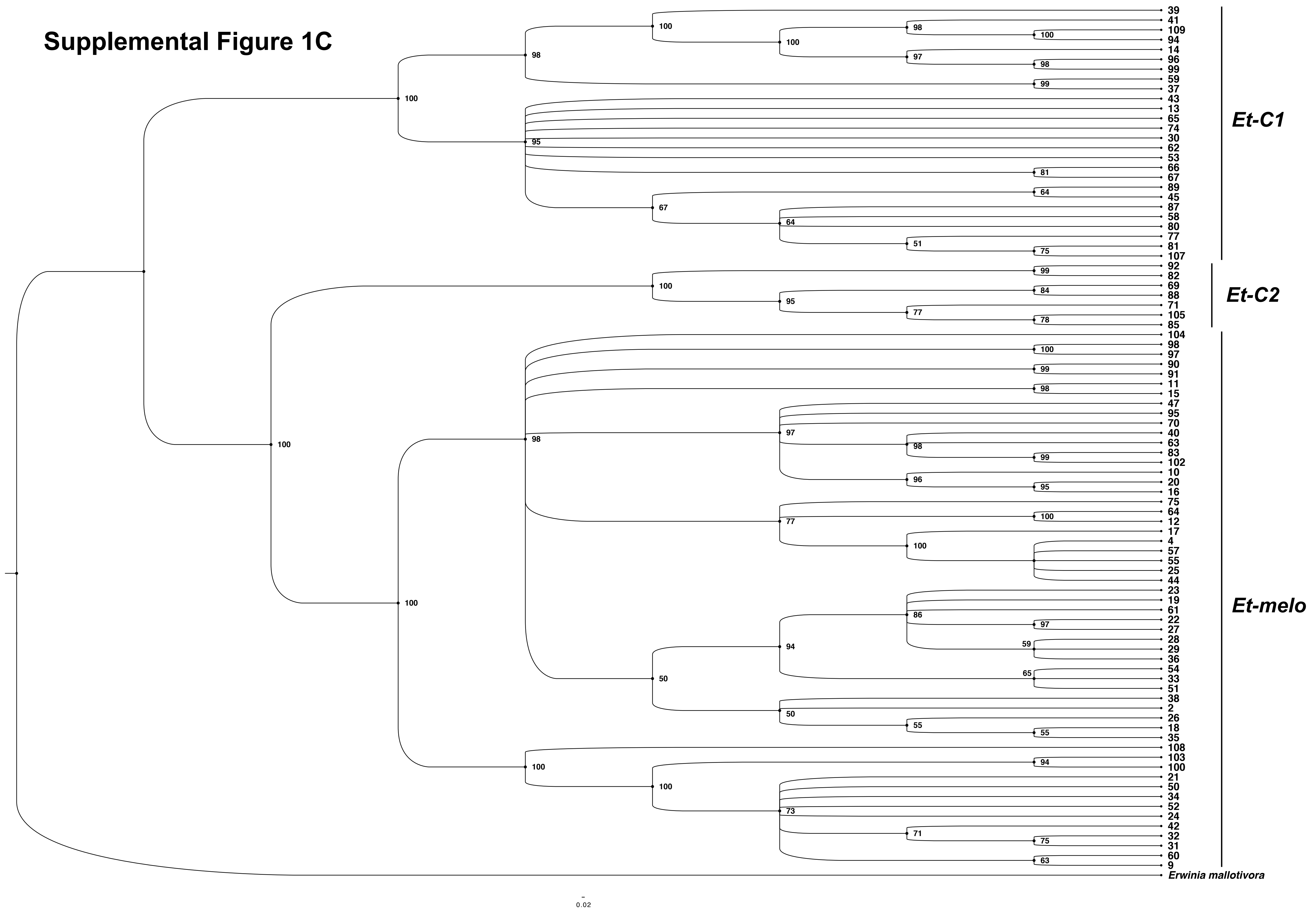

Supplement: FIG S1 [file mbo005184076sf1.pdf]

# AvrRpm1

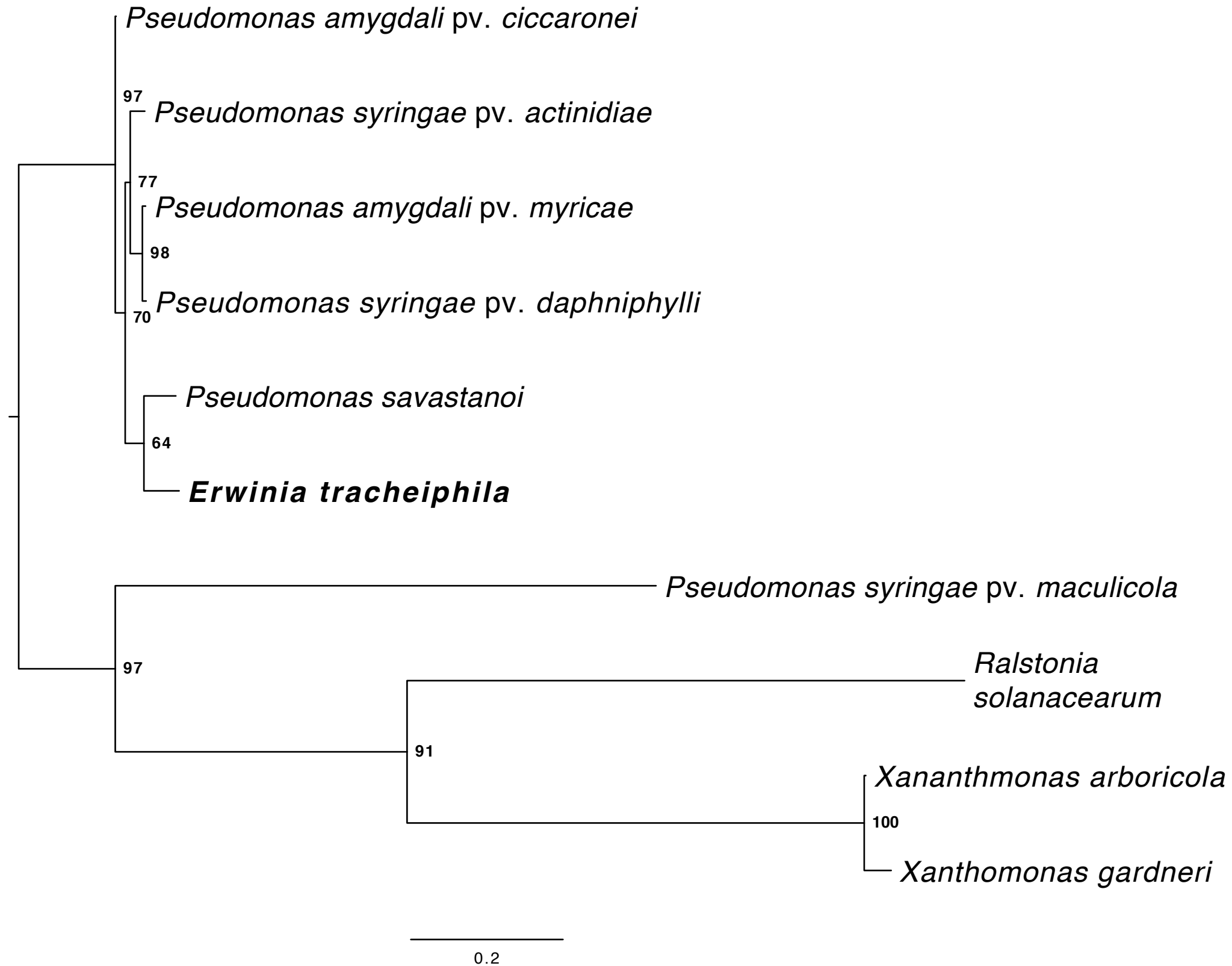

# HopAN1

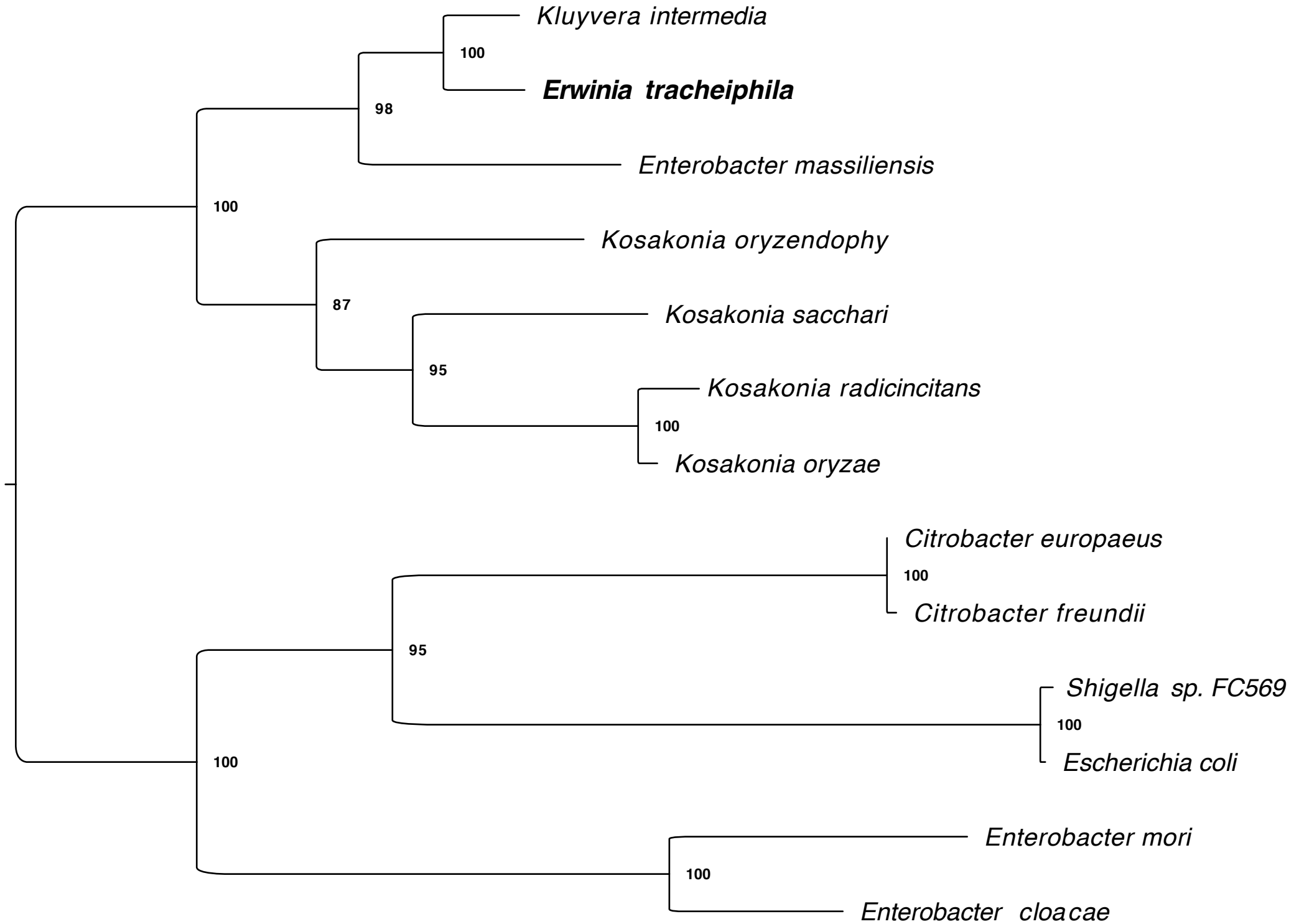

# Eop3

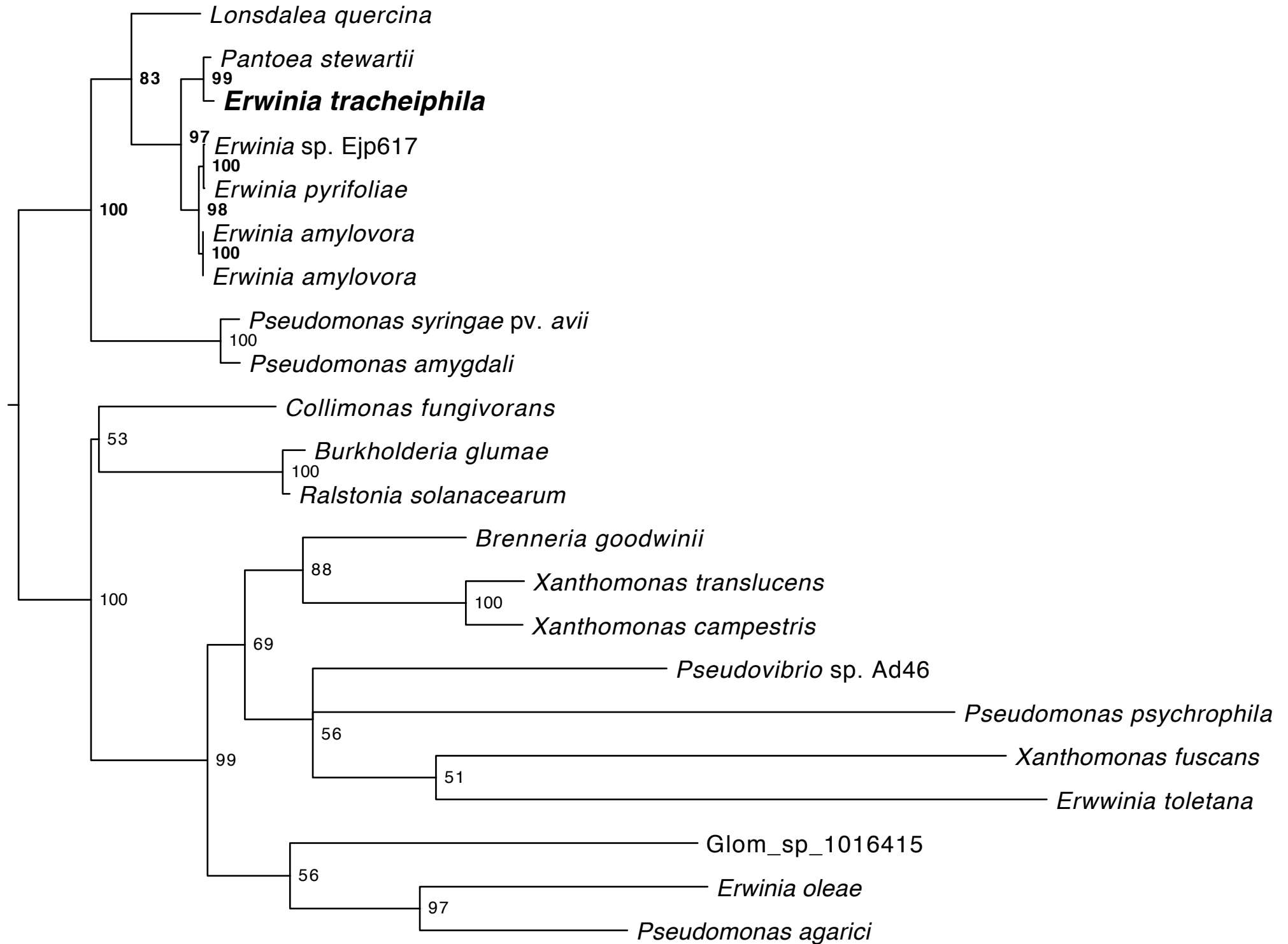

0.4

# NleD

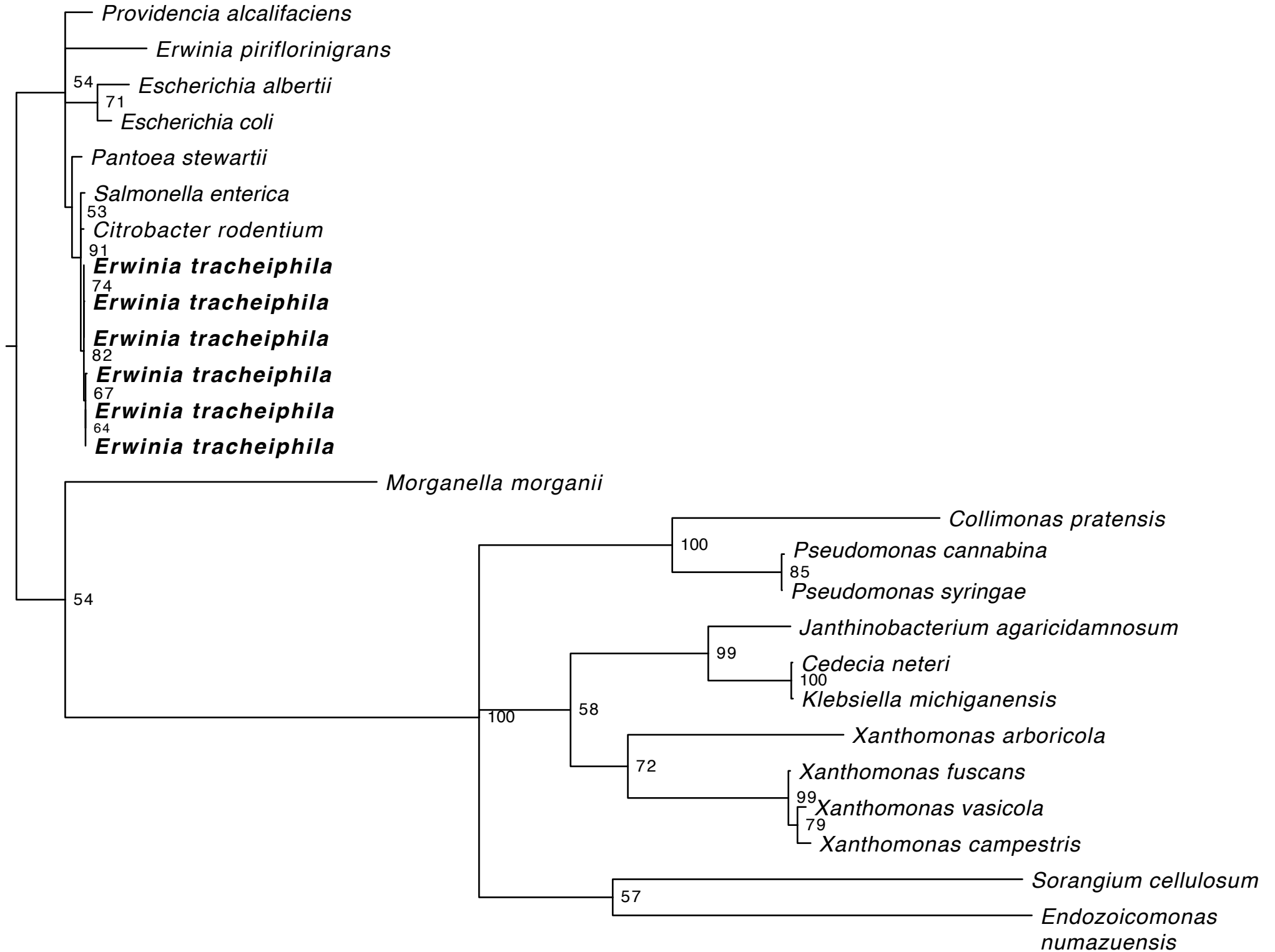

# HopAF1

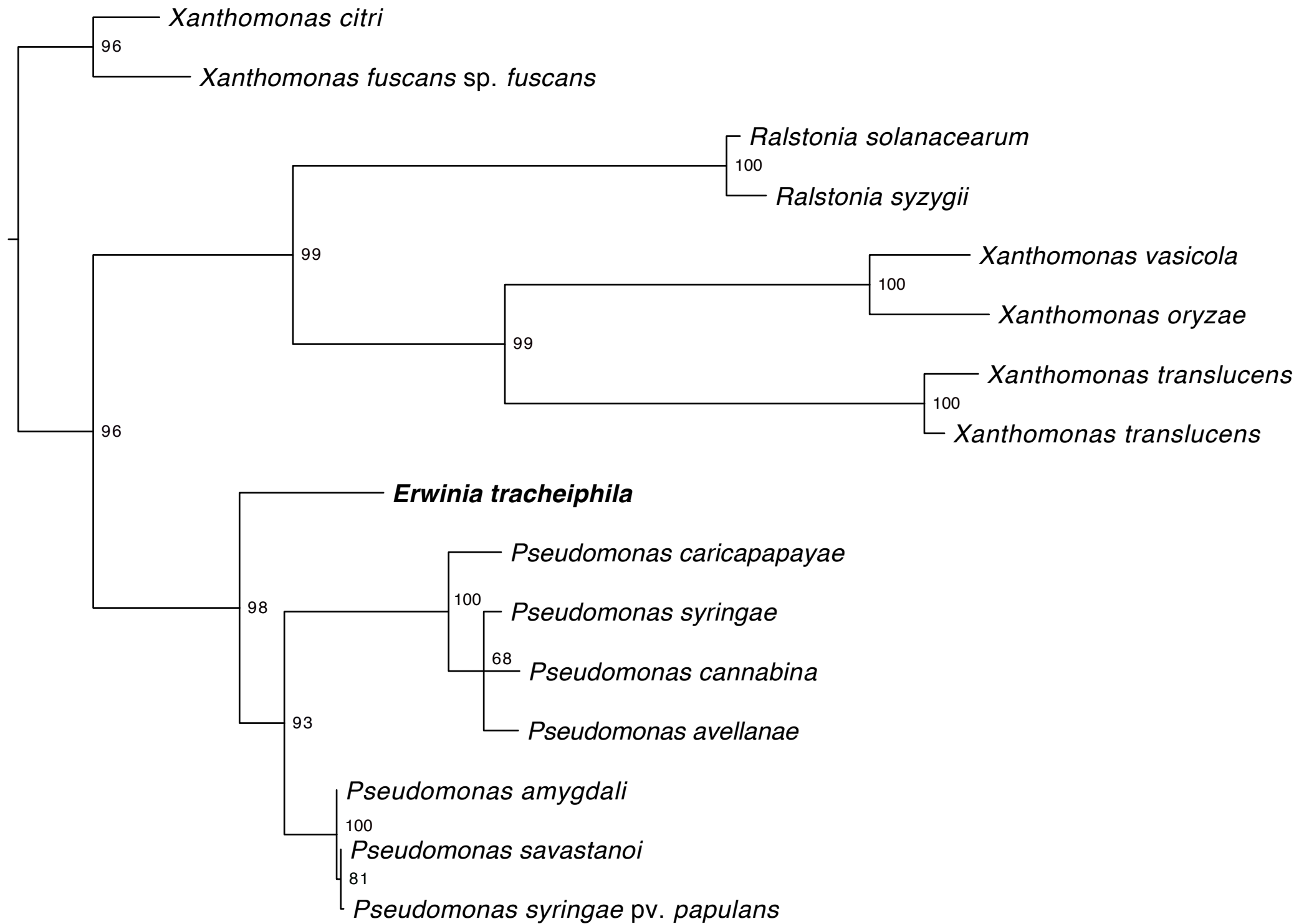

0.2

Supplement: FIG S2 [file mbo005184076sf2.pdf]
